# InSAR analysis reveals insights into the Ms 6.2 earthquake rupture and tectonic dynamics of the northeast margin of the Qinghai‒Tibet plateau

**DOI:** 10.1038/s41598-026-40753-7

**Published:** 2026-02-19

**Authors:** Guangtong Sun, Fenze Guo, Xinchen Guo, Minxue Li, Bo Peng

**Affiliations:** 1 Liaoning Technical University, Fuxin, 123000 China; 2 Institute of Disaster Prevention, Langfang, 065201 China; 3 Gansu Institute of Engineering Geology, Lanzhou, 730000 China

**Keywords:** 2023 Jishishan earthquake, InSAR, coseismic surface displacement, seismogenic structure, slip distribution, Natural hazards, Solid Earth sciences

## Abstract

On 18 December 2023, an Ms 6.2 earthquake occurred in Jishishan County, Linxia Prefecture, Gansu Province, China. As the strongest seismic event in the region since records began, it caused significant casualties and property damage while providing a valuable opportunity to investigate local seismogenic structures. In this study, the seismogenic structure and seismogenesis mechanism of earthquakes were explored through high-precision coseismic deformation analysis and fault modeling combined with regional tectonic background and aftershock distribution data. The results indicate that the earthquake was a typical shallow thrust event with a maximum slip of 0.7 m and a minor dextral strike-slip component. A small-scale dextral adjustment fault that developed at the intersection of Lajishan, the Yellow River, and Jishishan is presumably the primary controlling factor for the dextral component. This study revealed that the northeastern margin of the Qinghai‒Tibet Plateau achieves strain release through local block rotation, resulting in a unique plateau expansion mode. These findings are crucial for understanding the tectonic evolution at the frontal edge of the Himalayan collision zone and improving regional strong earthquake risk assessment.

## Introduction

**Fig. 1:**
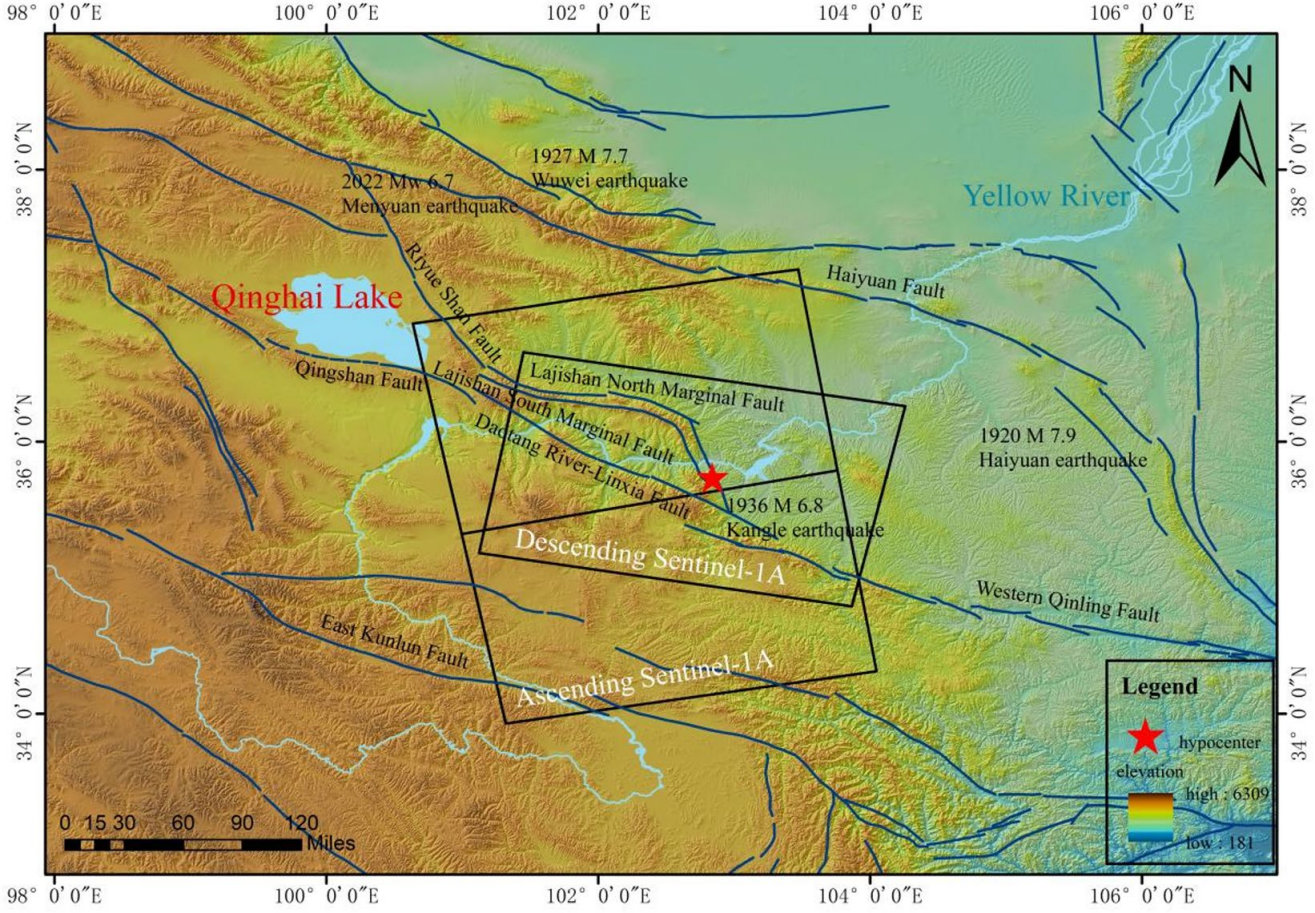
Tectonic background of the northeastern margin of the Qinghai‒Tibet Plateau and regional tectonic map near the Jishishan earthquake.

Owing to its complex strain distribution pattern, the northeastern margin of the Qinghai‒Tibet Plateau, a major active tectonic deformation front in Eurasia, has become a key research area for understanding the dynamics of intercontinental deformation^1^. This region is subject to the persistent north‒northeastward compression of the Indian Plate. Constrained by the combined resistance from the rigid Alashan and Ordos blocks, strain is concentrated in the Lajishan arcuate tectonic zone^2^. The Ms 6.2 Jishishan earthquake (Gansu Province, China), which occurred on 18 December 2023, represents the most intense seismic event recorded in this region to date. Given its strategic location at the structural transition between the Lajishan and Western Qinling tectonic belts (Fig. 1), this earthquake provides invaluable insights into the lateral expansion dynamics of the Qinghai–Tibet Plateau. The Lajishan tectonic zone, where the earthquake occurred, is a late Pleistocene arcuate mountain belt formed by compressional deformation and uplift along the northeastern margin of the Qinghai‒Tibet Plateau^3^. The western segment of this zone is contiguous with the dextral strike-slip Minyue Mountain Fault Zone, whereas its eastern segment abuts the sinistral strike-slip Western Qinling Tectonic Belt. This geographical and structural setting renders the Lajishan tectonic zone a critical compressional tectonic domain and structural transition zone, mediating stress and strain transfer between these two distinct fault systems^4,5^. The regional geological structure in the vicinity of the Jishishan earthquake is illustrated in Fig. 1.

Following the earthquake, numerous scholars have researched the seismogenic structure; however, debates regarding the structural characteristics and seismogenic mechanism of the seismogenic fault remain ongoing. Wang, Z., et al.^6^ employed nonlinear Bayesian methods and linear inversion methods to confirm that the seismogenic fault of this earthquake was a thrust fault and left-lateral strike-slip fault at the southeastern end of the northern margin fault of Lajishan, which verified the northeast-dipping nodal plane as the seismic rupture plane. Zhou, M., et al.^7^ reported that after the earthquake, the Coulomb failure stress significantly increased in certain segments of the Qinghai Nanshan–Xunhua Nanshan Fault, as well as the northern and southern margin faults of Lajishan. Notably, the increase in stress in the eastern segment of the Qinghai Nanshan–Xunhua Nanshan Fault far exceeded the static stress triggering threshold. An analysis of three subsequent larger aftershocks indicated that the Ms 6.2 Jishishan earthquake exerted a substantial triggering effect. Ping, Z., et al.^8^ reported that the seismogenic fault of the 18 December 2023 Ms 6.2 Jishishan earthquake (Gansu Province) is a concealed fault with NW–SE strike and SW dip, potentially a branch fault of the Eastern Margin Fault of Lajishan, and classified the earthquake as a thrust and right-lateral strike-slip event. Cai, G., et al.^9^ revealed the three-dimensional velocity structure and seismogenic structure of the source area using the double-difference tomography method. Their research suggests that the northeast-dipping Dahejia Fault is the seismogenic fault responsible for the mainshock, whereas the aftershock belt north of the main rupture is likely located on a southwest-dipping back-thrust blind fault. The primary deep tectonic factor controlling the distribution of mainshocks and aftershocks is the heterogeneity of the velocity structure: earthquakes are typically concentrated in areas with high-velocity and low-Poisson’s ratio anomalies, a phenomenon that reflects the process of stress accumulation and release. Additionally, Chen, X., et al.^10^ compiled the focal mechanism solutions of the 2023 Ms 6.2 Jishishan earthquake sequence (Gansu Province) and performed fuzzy cluster analysis. The results reveal that the region is subjected to eastward compression from the Qinghai‒Tibet Plateau, resulting in a southwest–northeast-trending compressional and southeast–northwest-trending extensional stress state. Furthermore, the two types of principal nodal planes of the earthquake are primarily distributed in areas characterized by high shear stress.

Notably, traditional seismic dislocation inversion predominantly relies on the Okada elastic half-infinite space analytical model, which assumes a homogeneous medium and thus fails to account for the effects of topographic relief and medium heterogeneity in the source area^11–13^. Recently, Chen et al.^14^ proposed an InSAR-constrained parallel elastic finite element model that incorporates medium heterogeneity characteristics via numerical Green’s functions, thereby effectively improving the accuracy of coseismic fault dislocation inversion. Tests based on the ideal models in this study demonstrated that a mere 10% difference in the Young’s modulus across the fault interface can significantly affect the inversion results. Moreover, the effective capture of medium heterogeneity signals can substantially reduce inversion nonuniqueness and location errors, providing important methodological insights for research on seismogenic structures. Unlike research approaches that directly integrate 3D velocity structures, in this study, a forward model of the 3D seismic deformation field was constructed, the 1D InSAR line-of-sight (LOS) deformation was decomposed into three-dimensional displacement components (vertical, eastward, and northward), and the differences in deformation responses induced by medium heterogeneity were indirectly captured. This approach thereby compensates for the limitations of the homogeneous medium assumption inherent in the traditional Okada model.

To improve our understanding of the seismogenic structure, seismogenic environment, and interaction between the aftershock sequence and deep structures of this seismic event, this study systematically integrated multisource data, including the coseismic deformation field derived from InSAR technology, seismological inversion results, and regional geological structural settings. These datasets collectively clarify the geometric characteristics of the seismogenic fault and the kinematic parameters associated with the earthquake. Concurrently, on the basis of the regional structural background and the spatial distribution of aftershocks, we investigated the seismotectonic model and its underlying dynamic mechanisms. This research aimed to provide a solid scientific basis for earthquake prediction and the formulation of disaster prevention and mitigation strategies in this region.

## InSAR data processing

### Data source

In this study, C-band (wavelength ~ 5.6 cm) Sentinel-1 A ascending and descending track satellite radar images, which were downloaded from the European Space Agency (https://dataspace.copernicus.eu/), were used to process differential interferometric data related to the 2023 Jishishan Earthquake. This enabled the extraction of the coseismic line-of-sight (LOS) surface displacement field associated with the earthquake. Detailed information regarding the SAR images is provided in Table 1, while Fig. 1 illustrates the coverage range of these images. The D-InSAR module within SARScape software was employed to process the Sentinel-1 data from both the ascending tracks and the descending tracks. Furthermore, in this study, 30-m spatial resolution SRTM-DEM data were used to mitigate topographic effects, and precise orbit data released by the European Space Agency were incorporated for orbit correction. To model earthquake deformation, the source mechanism published by the Global Centroid Moment Tensor Project (GCMT) was adopted as the initial source parameter (accessed from https://www.globalcmt.org/CMTsearch.html).

**Table 1:** Information on the data used in this study.

Satellite	Orbit	Reference Date	Repeat Date	Vertical Baseline	Incident Angle	Azimuth Angle
Sentinel-1 A	Ascending track128	20,231,027	20,231,226	64	41.5	-9.9
Sentinel-1 A	Descending track135	20,231,214	20,231,226	-117	39.4	-169.9

### Data processing procedure

**Fig. 2:**
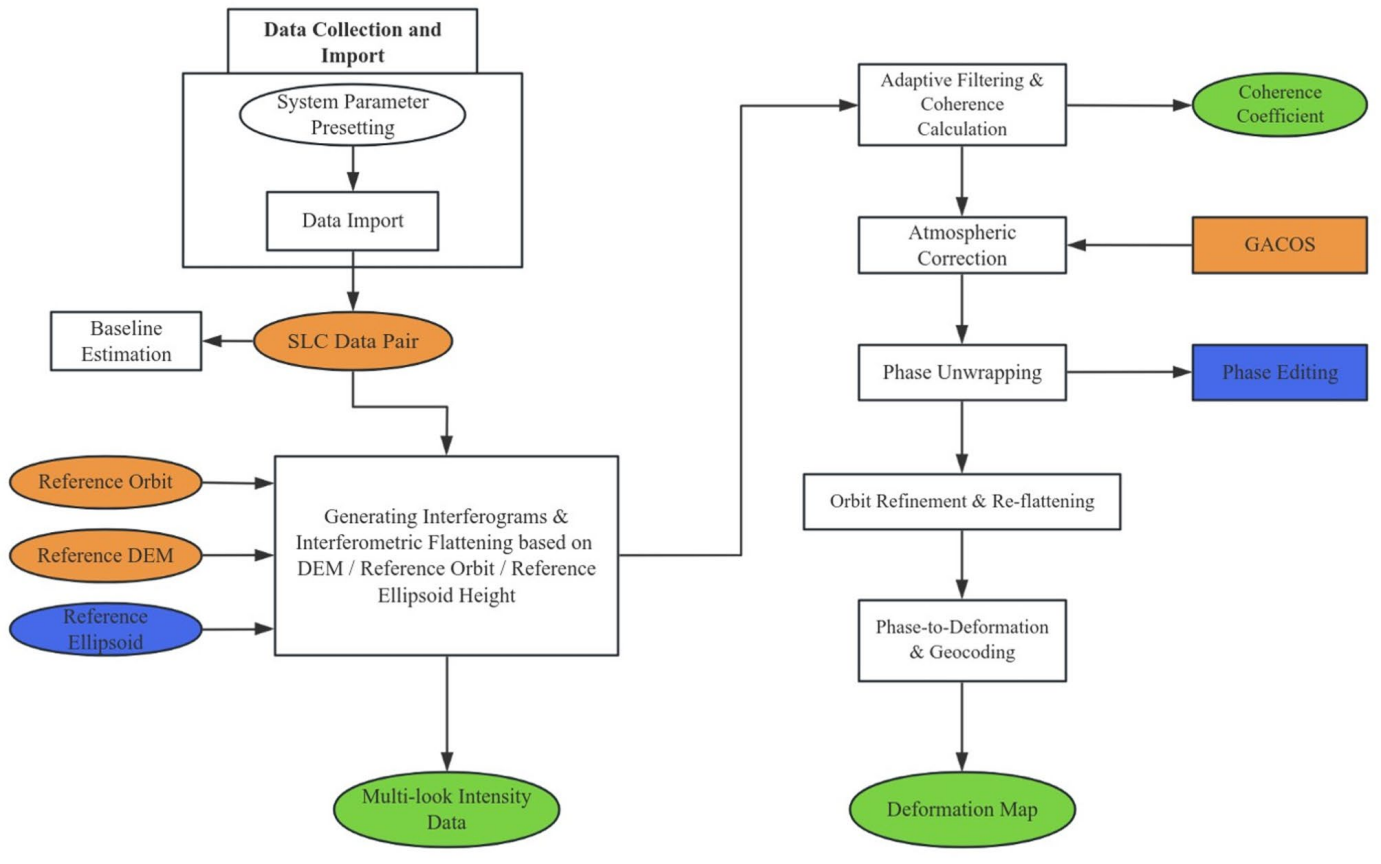
InSAR data processing flowchart.

In the data processing stage, the primary focus was on Sentinel-1 images with VV polarization. Initially, a connection graph was constructed, and the selected images were automatically registered to the optimal master image. To improve the signal-to-noise ratio (SNR), multilooking processing was performed with a multilook factor of 10 in the range direction and 2 in the azimuth direction. The Goldstein adaptive filter was applied for interferometric phase filtering. To mitigate atmospheric delay effects, GACOS data were used for atmospheric correction of the interferometric results. In the generated interferograms, the phase varies periodically between -π and π; when the actual phase value exceeds π, it restarts from -π and repeats with a 2π cycle. To retrieve the actual phase value from the differential phase, pixels whose coherence was lower than 0.15 were masked out. The minimum cost flow (MCF) algorithm was employed for wrapped phase unwrapping. To optimize the phase unwrapping results, a set of ground control points (GCPs) representing stable areas was used for polynomial fitting. Subsequently, the interferograms and unwrapped phases underwent orbit refinement and reflattening to recover the LOS surface displacement. Finally, the obtained LOS coseismic displacement field was geocoded using DEM data to generate coseismic deformation results under the WGS84 coordinate system. The data processing workflow is illustrated in Fig. 2.

## Coseismic deformation results

**Fig. 3:**
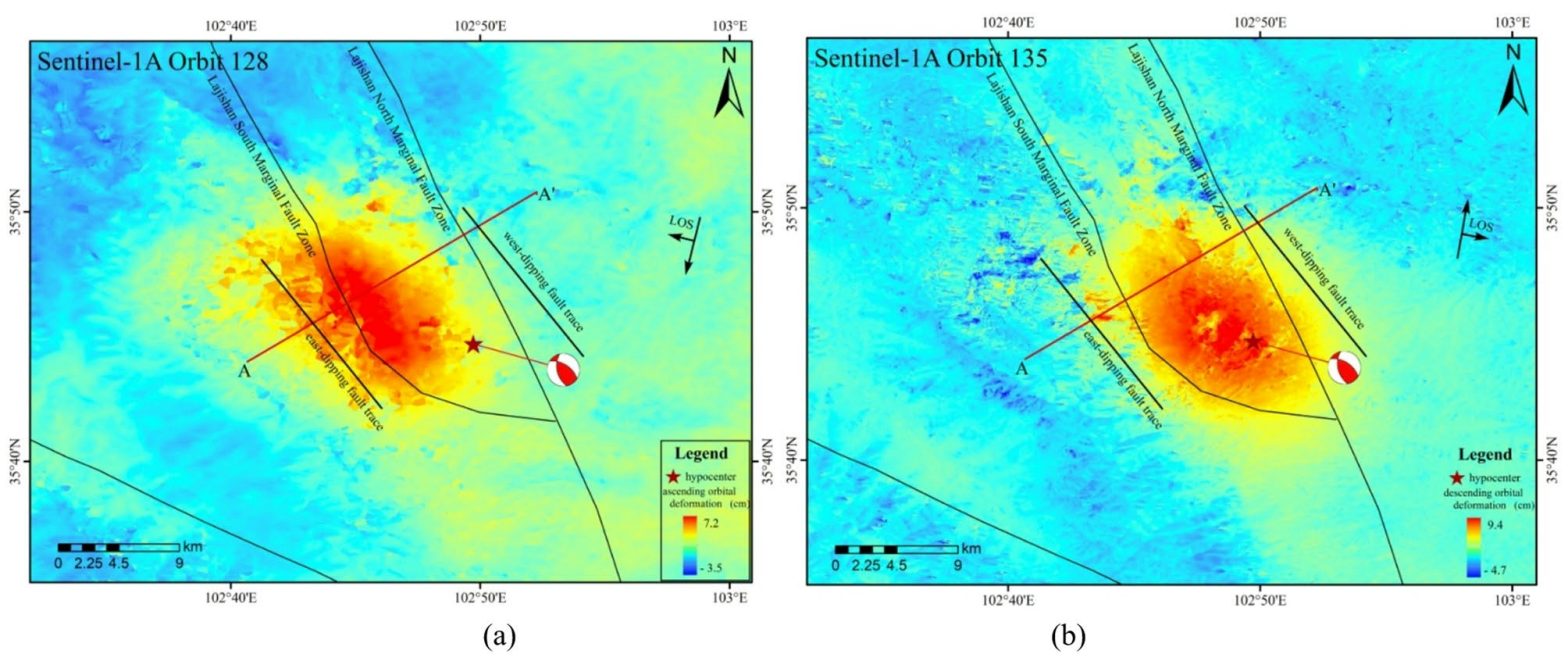
Coseismic surface deformation field of the 2023 Jishishan earthquake. (**a**) Ascending track coseismic deformation field, (**b**) Descending track coseismic deformation field.

The coseismic deformation outcomes of the 2023 Jishishan earthquake, as analyzed through InSAR, are shown in Fig. 3. The spatial characteristics of this earthquake deformation reveal that the primary deformation area is situated between the northern and southern margin faults of Lajishan. The elliptical distribution suggests that the seismogenic fault extends in a northwest–southeast (NW–SE) direction. The coseismic surface deformation area spans approximately 15 km by 25 km. The maximum uplift along the line-of-sight (LOS) direction in the ascending track is approximately 7.2 cm, whereas the maximum subsidence is -3.5 cm. Conversely, the maximum uplift along the LOS direction in the descending track is approximately 9.4 cm, with a maximum subsidence of -4.7 cm. Owing to the extended time baseline of the ascending track images, temporal decorrelation resulted in minor signal loss in the western portion of the deformation field.

**Fig. 4:**
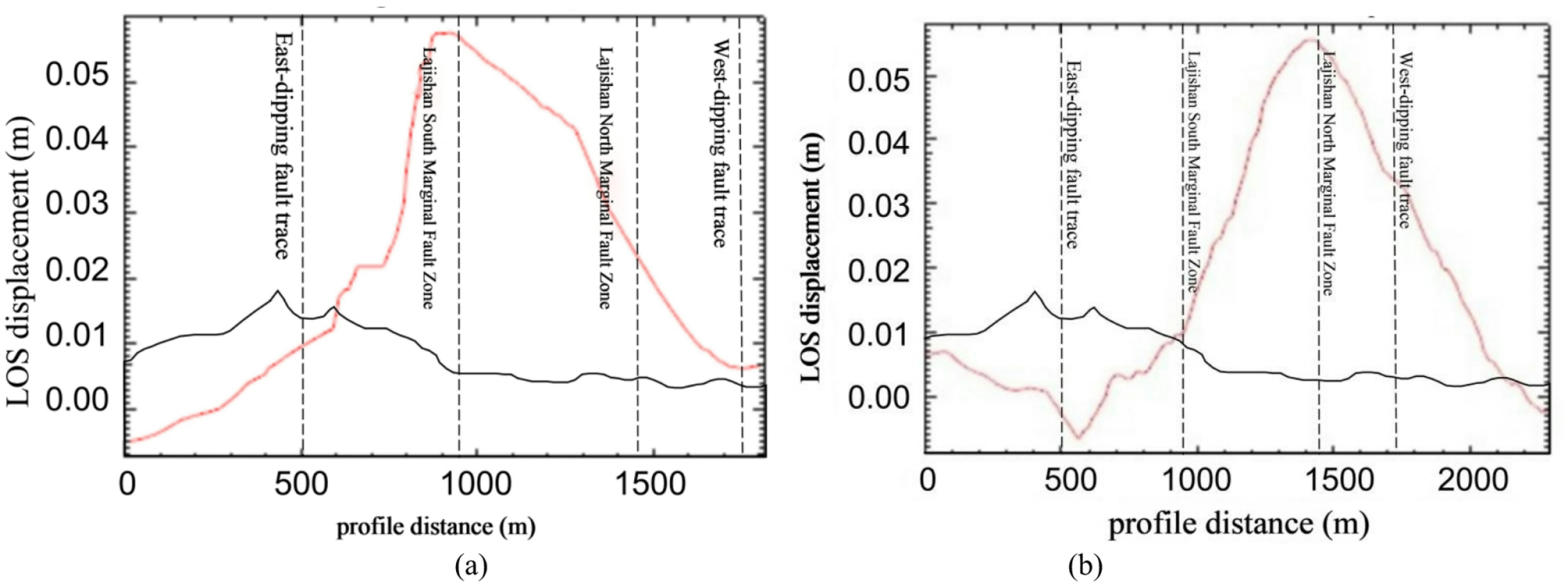
Surface deformation profile of the 2023 Jishishan earthquake. (**a**) Cross-fault profile derived from the ascending track deformation field, (**b**) Cross-fault profile derived from the descending track deformation field.

Within the InSAR deformation field, the 1-km-wide cross-fault profile line AA’ was selected (refer to Fig. 3), and its corresponding profile map is presented in Fig. 4. Analysis of the coseismic deformation field, in conjunction with the profile map, reveals that the deformation values in the epicentral area share the same sign, indicating universal shortening toward the image. These observations confirm a distinct uplift center, suggesting that the deformation induced by this earthquake was primarily vertical. Furthermore, the profile displacement curve is continuous and smooth and lacks displacement steps, which could be attributed to abrupt changes in the deformation gradient. This further indicates that the seismogenic fault did not rupture to the surface.

## Focal mechanism interpretation

After the coseismic deformation field was derived, the Okada elastic half-space dislocation model^11,12^ was used to infer the fault geometry and slip distribution of the seismogenic fault. The first step involved generating a sampling area on the basis of the coseismic deformation results, with the quadtree algorithm employed for image downsampling. Given the high spatial correlation of observations due to the excellent spatial resolution of InSAR data, the ascending track deformation results were prioritized as the modeled source data. This approach was adopted to improve the computational efficiency of the inversion process and minimize the impact of far-field noise on the inversion results, resulting in a larger sampling area for the ascending track than for the descending track. A sampling interval of 500 m was established in the inner deformation zone, whereas the interval was set to 2000 m in the outer zone.

In this study, the combined uplift and subsidence deformation results were used as the observational dataset, which was further processed to calculate the observed displacements for use as the predicted geodetic source data. These calculations were based on a suite of geophysical seismic source parameters retrieved from the GCMT website. The solutions provided by the centroid moment tensor (CMT) include only point source parameters such as strike, dip, slip angle, source location and depth, and seismic moment. The fault plane dimensions (length and width) and slip magnitude can be inferred from these parameters using the empirical formula proposed by Wells & Coppersmith^13^. On the basis of this formula, the fault plane length is 14,299.1 m, the width is 8,709.6 m, and the slip magnitude is 0.2539 m.

### Fault geometric parameter inversion

**Fig. 5:**
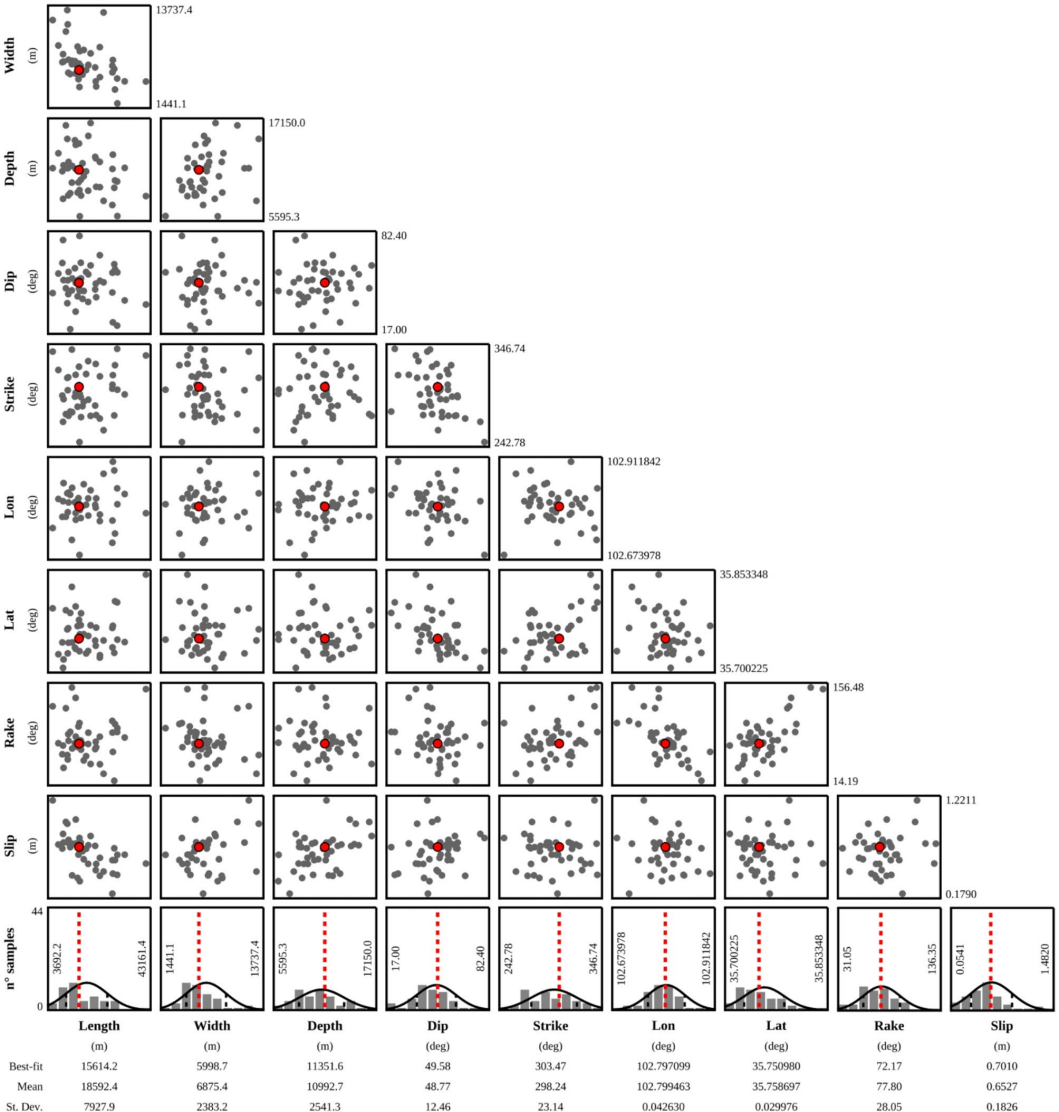
Marginal posterior probability distributions of seismogenic fault parameters for the 2023 Jishishan earthquake; subplots show the joint probability distributions of relevant parameters.

Given that the forward modeling process of the seismic source deformation field (i.e., calculating surface deformation from seismic source parameters) exhibits significant nonlinear characteristics, linear inversion methods are inadequate for accurately characterizing the complex correlation between observed data and seismic source parameters. Consequently, in this study, a nonlinear inversion approach was employed to determine the optimal seismic source parameters. The core of this approach lies in the Levenberg–Marquardt (L-M) nonlinear least-squares optimization algorithm^15,16^, which effectively balances the iterative convergence rate and solution stability. In the nonlinear inversion process, the deformation source is treated as a priori unknown, and each source parameter can be directly inferred from geodetic data. Since the reference point in the interferogram may be situated within a deformed zone, we modeled the data using a single-fault model while incorporating potential offsets that could affect the data. During the inversion, iterative computations were performed using the L-M least-squares optimization algorithm on the basis of the discrepancy between observed and predicted geodetic data, yielding the optimally fitted solution for the fault parameters. As illustrated in the joint probability density function (PDF) distribution of the source parameters (Fig. 5), all the parameters approximated a normal distribution and exhibited low pairwise correlation—validating both the stability of the inversion results and the independence of the parameters.

Table 2 illustrates the focal mechanism solutions provided by various institutions and studies. The earthquake inversion focal mechanism presented in this paper is closely consistent with those provided by other research institutions and scholars, thereby verifying the credibility of the research in this paper.

**Table 2:** Source parameters of the 2023 Jishishan earthquake in Gansu.

Hypocenter	Longitude (°)	Latitude (°)	Strike (°)	Dip (°)	Slip (°)	Depth (km)	Magnitude (Mw)
This study	102.79	35.75	303.47	49.59	0.7	11.35	6.0
USGS	102.82	35.74	333	52	62	10	5.9
CENC	102.78	35.7	-	-	-	10	6.2(Ms magnitude)
Yang, J., et al.^18^	102.92	35.71	311	53.8	79.6	6.6	6.1
Liu, Z., et al.^19^	102.81	35.76	319	42	-	9.3	6.0
Fang, N,. et al.^20^	102.76	35.77	325	32	112	7.7	6.0

### Distribution of the coseismic fault slip

After determining the fault geometric parameters via nonlinear inversion^15,16^, this study utilized the Okada elastic dislocation model to conduct linear inversion analysis of the fault slip distribution^17^, aiming to accurately capture the characteristics of the slip field on the fault plane. To improve the inversion precision, the target fault plane was first spatially extended and then structurally discretized. Specifically, it was extended 30 km along the fault strike and 20 km in the downdip direction, resulting in a discretized fault model consisting of 600 regular grid blocks (1 km × 1 km each). By utilizing InSAR deformation observation data from both the ascending tracks and the descending tracks, an equal-weight joint inversion strategy was adopted to construct the observation equation. The optimal solution for the slip distribution parameters was obtained through matrix operations.

**Fig. 6:**
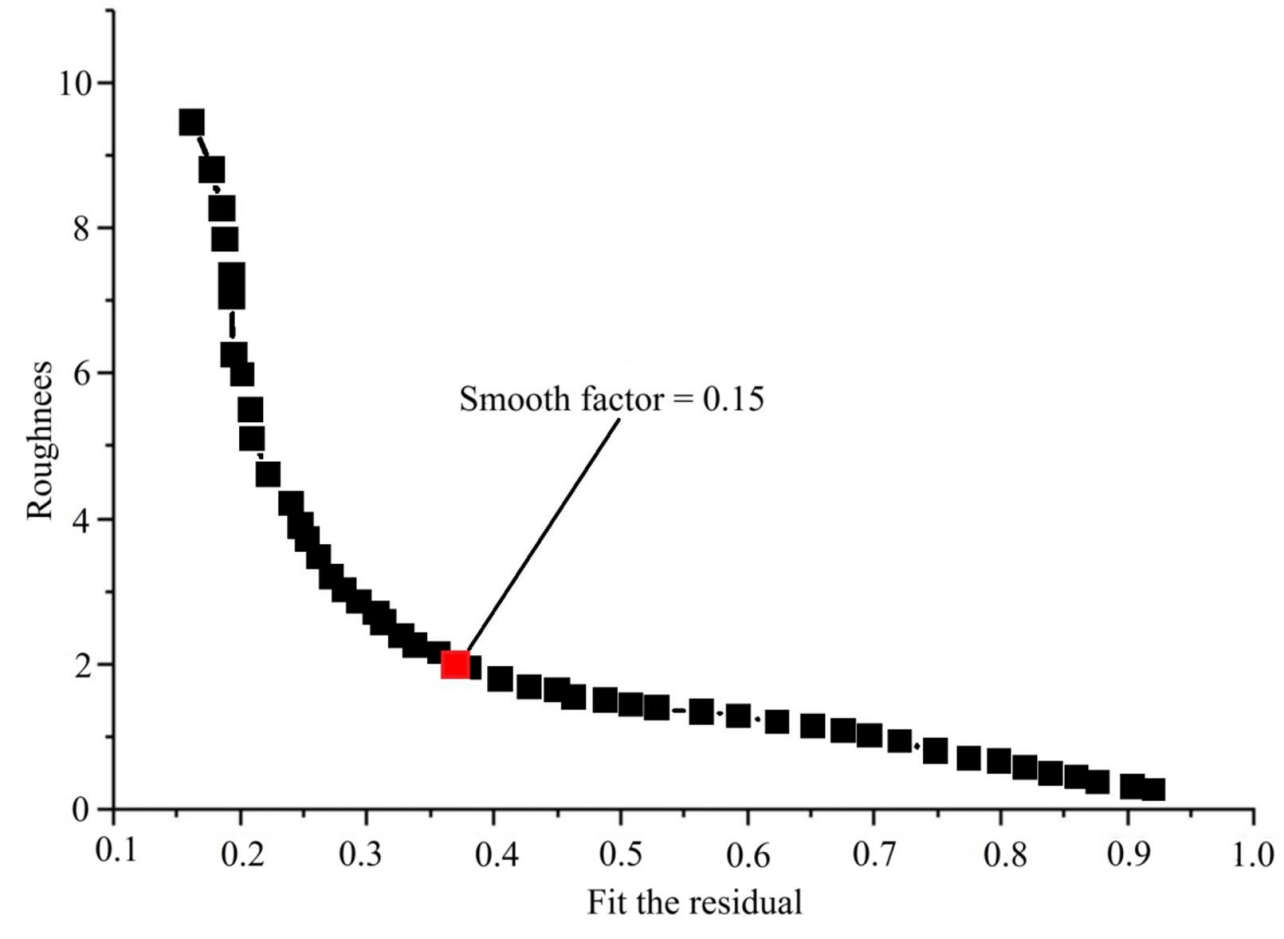
Comparison curve between fault plane slip roughness and fitting residuals for the 2023 Jishishan earthquake inversion; the compromise position was selected to determine the smoothing factor (0.15) for applying fault slip smoothing constraints.

The Tikhonov regularization method was employed for the linear inversion regularization process^21^, with the damping factor serving as the primary parameter to adjust the resolution and stability of the model. Its value was determined by comprehensively balancing the fitting residual and the smoothness of the solution, guided by the L-curve criterion^22^(Fig. 6). After multiple tests to balance the fitting residual and roughness, a final compromise damping coefficient of 0.15 was selected. This effectively suppressed the instability of the solution while preserving the primary slip characteristics.

**Fig. 7:**
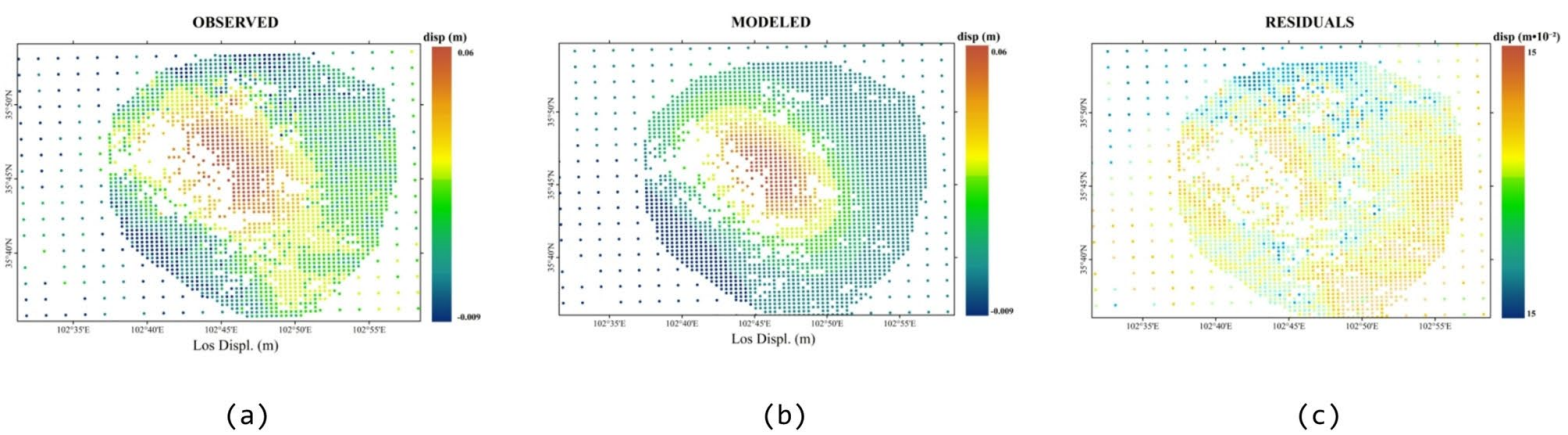
Comparison of the ascending track InSAR-observed deformation and Okada model simulation results and residual distribution. (**a**) Ascending-track observed deformation field, (**b**) Ascending-track simulated deformation field, (**c**) Residual field between ascending-track observation and simulation.

**Fig. 8:**
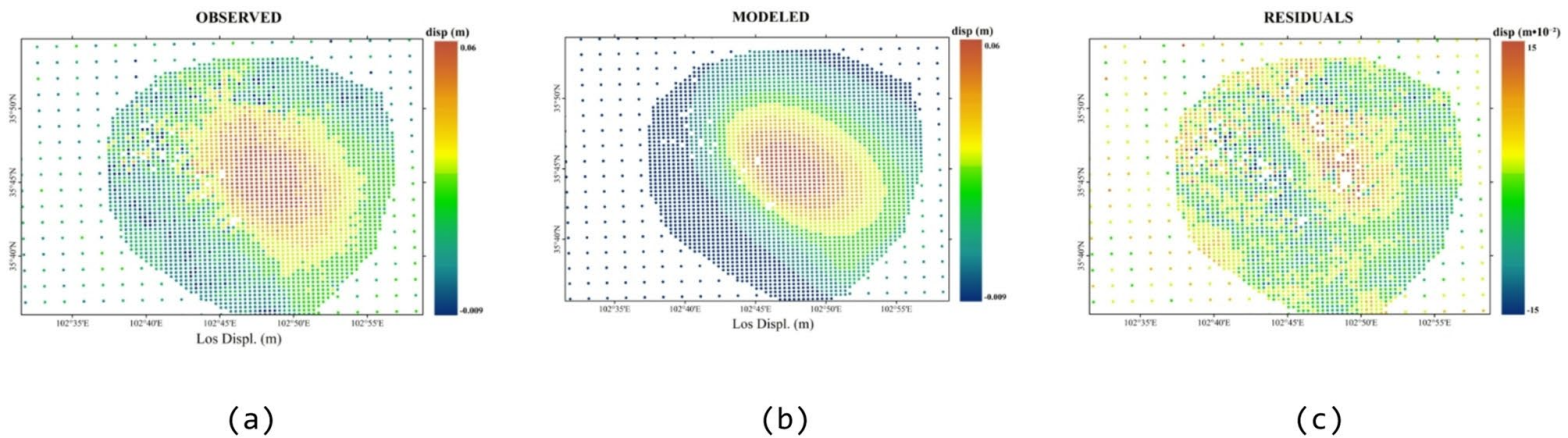
Comparison of the descending-track InSAR-observed deformation and Okada model simulation results and residual distribution. (**a**) Descending-track observed deformation field, (**b**) Descending-track simulated deformation field, (**c**) Residual field between descending-track observation and simulation.

The findings from the ascending and descending track inversions are presented in Figs. 7 and 8, respectively. The integrated inversion results exhibit significant spatial consistency with the InSAR-observed deformation field. The root mean square (RMSE) residuals for both the ascending track data and the descending track data remained stable at 0.006 m, representing an order of magnitude reduction compared with the original observed values. These significant differences comprehensively validate the earthquake fault inversion results detailed in this study, underscoring the effectiveness of the proposed elastic dislocation inversion framework in accurately capturing the spatial heterogeneity characteristics inherent in fault activity.

**Fig. 9:**
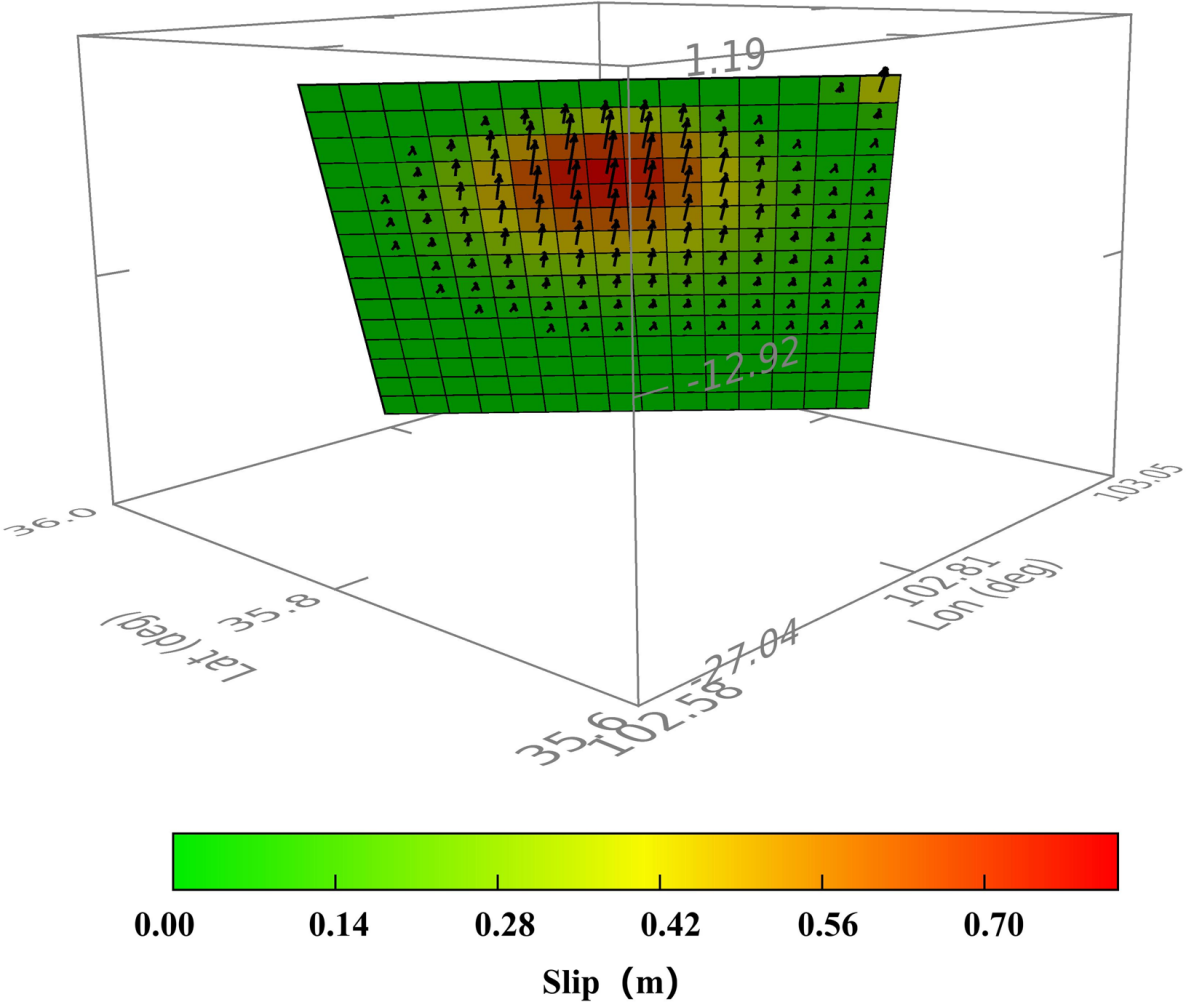
Inversion results of the fault slip distribution for the Jishishan earthquake.

The detailed slip distribution characteristics of this seismic event are presented in Fig. 9. Comprehensive inversion results revealed that the earthquake was a thrust-type shallow tectonic event, with the seismogenic fault exhibiting a geometric morphology dipping northeast at 49.6° and striking northwest at 303.5°. Slip was predominantly concentrated in the lower segment of the fault plane, spanning approximately 15.6 km along the strike and 0.6 km along the dip, forming a semielliptical shape. The maximum recorded coseismic displacement was 0.7 m, and the slip depth was primarily concentrated in the 0–15 km subsurface range, with the hypocenter located at a depth of 11.4 km.

Compared with those of strike-slip and normal fault earthquakes, the rupture mechanism of thrust faults has a significantly greater impact on surface deformation^23^. In this event, the unusually shallow coseismic slip directly led to energy release near the surface. This characteristic of shallow fault displacement resulted in amplified seismic wave amplitudes close to the surface. Combined with the distinct vertical displacement component of thrust earthquakes, these factors contributed to the disaster, causing significant casualties and infrastructure damage.

## Discussion on seismogenic structure

**Fig. 10:**
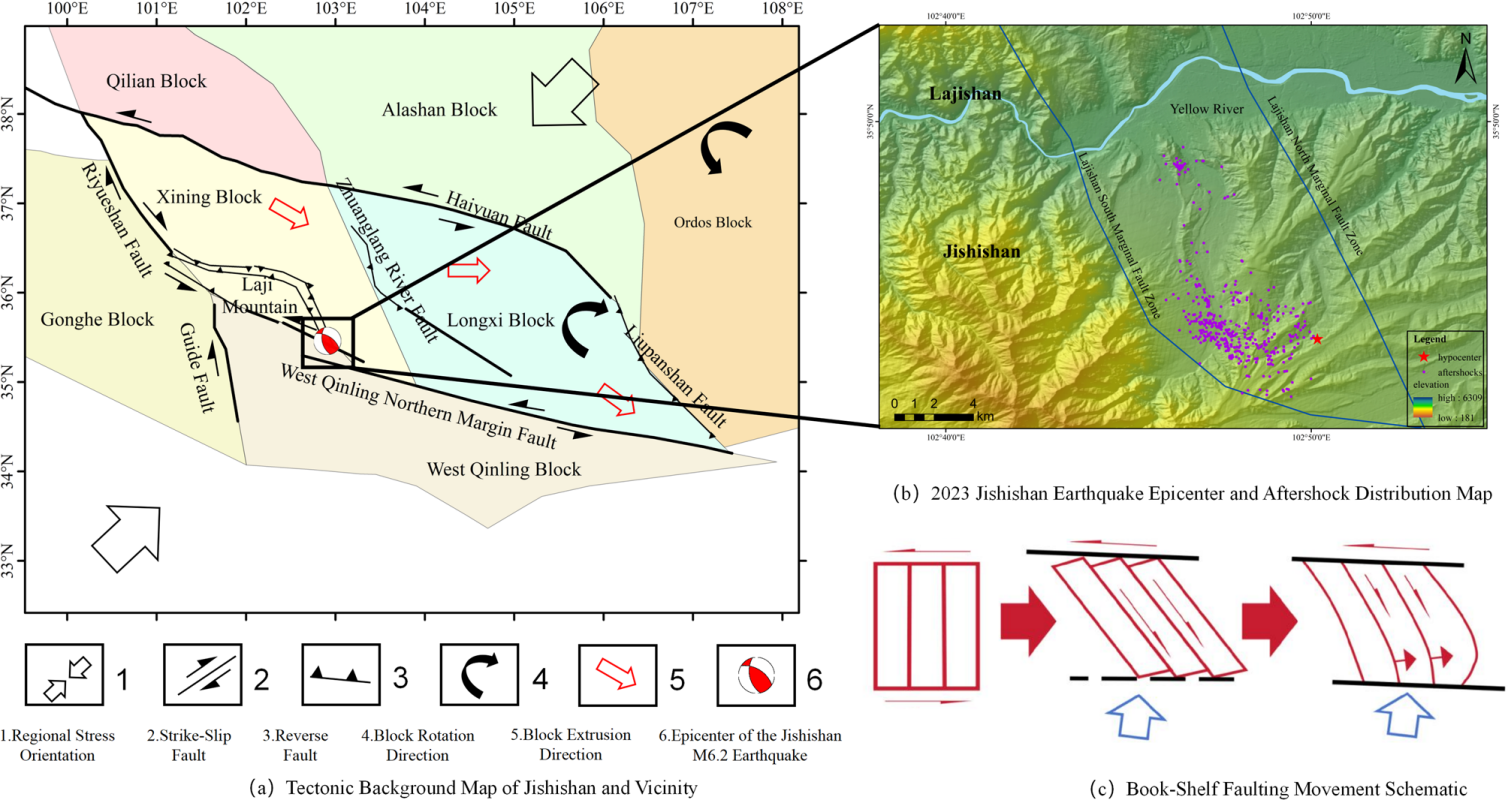
Tectonic activity pattern map of the area near Lajishan: (**a**) Tectonic background map of Jishishan and its vicinity; (**b**) map of the 2023 Jishishan earthquake epicenter and aftershock distribution; (**c**) schematic of the bookshelf fault movement.

Analysis of the regional tectonic background (Fig. 10(a)) revealed that the Jishishan seismic sequence occurred at the northeastern margin of the Qinghai‒Tibet Plateau, driven by the continuous northeastward (NE) indentation of the Indian Plate. The Lajishan fault zone is influenced by the differential slip rates of the northern Haiyuan Fault and the southern northern margin fault of the Western Qinling^24^. Concurrently, owing to the combined blocking effect of the Alashan Block^25^ and the rigid Ordos Block, the Xining–Lanzhou secondary block underwent southeastward (SE) clockwise rotational extrusion deformation, dominated by a north‒northeastward (NNE)-trending principal compressional stress field^26^. This unique strain partitioning mechanism gave rise to the formation of a prominent compressional thrust belt at the termination of the strike-slip fault zone^27^.

**Fig. 11:**
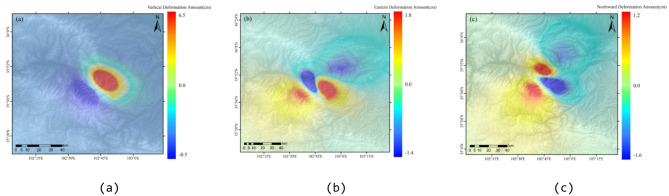
Spatial distribution of 3D coseismic displacement components of the Jishishan earthquake. (**a**) Vertical deformation variable, (**b**) Eastward deformation variable, (**c**)Northward deformation variable.

To address the inherent limitations of D-InSAR technology—namely, its ability to acquire only one-dimensional line-of-sight (LOS) deformation observations—a forward model of the 3D seismic deformation field was constructed. This model takes the inverted fault slip distribution parameters as its core input, the geophysical source parameters provided by the global CMT as macroscopic dynamic constraints, and the surface LOS deformation values observed by D-InSAR as surface response constraints. Among the global CMT-derived geophysical source parameters, the focal depth clarifies the rupture initiation position to optimize the model’s geometric boundaries; the seismic moment tensor inverts the total seismic deformation energy and equivalent slip magnitude to constrain the slip range; and the focal mechanism solution further limits the dominant slip direction. On the basis of the Okada elastic half-space mechanical model, the forward-calculated 3D displacement components (vertical, northward, and eastward) are projected onto the radar line-of-sight direction, the forward-simulated values are compared with the D-InSAR observed values, and parameters such as the local fault slip magnitude and slip boundaries are iteratively optimized to minimize the residuals. By integrating subsurface fault slip information with surface deformation observation data, the model can fit the actual seismogeological process, thereby overcoming the limitations of one-dimensional observations and achieving reliable calculations of the earthquake’s 3D seismic deformation field.

The results of the forward calculation (Fig. 11) revealed that the coseismic deformation field is characterized primarily by vertical displacement (maximum of 6.5 cm), supplemented by the spatial distribution of the eastward component (1.8 cm) and the northward component (1.2 cm). These findings clearly reflect the dynamic process of strain adjustment, which occurs through vertical crustal thickening under the continuous NNE-induced compression of the Indian Block on the northeastern margin of the Qinghai‒Tibet Plateau. Notably, this earthquake was strictly controlled by the Jishishan piedmont tectonic zone south of the Yellow River, while no significant aftershocks were detected in the Lajishan piedmont east of the Yellow River (Fig. 10(b)). Geological structure analysis revealed that Lajishan and Jishishan are deeply separated by the Yellow River, with obvious discontinuities in the mountain boundaries and slope fold belts. The dextral displacement component is concentrated on the south side of the Yellow River valley. This tectonic pattern suggests the possibility of a NNW-trending dextral adjustment fault at the intersection of the Lajishan-West Qinling Fault. The activity of this fault would balance the differential deformation between boundary fault strike-slip and block thrusting.

In the southern portion of the Lajishan fault zone, eastward displacement indicates dextral strike-slip movement. This movement is not isolated and may be significantly associated with the remote shear effect of the Western Qinling sinistral fault zone. Specifically, under the influence of large-scale sinistral boundary faults, NNW-trending fractures form within the crust, segmenting it into multiple small blocks. These secondary blocks undergo book-shelf faulting under simple shear (as depicted in Fig. 10(c)). The concentration of aftershocks on the south side of the Yellow River valley (refer to Fig. 10(b)) further corroborates the existence of NNW-trending dextral adjustment faults. These faults absorb the torque generated by rotation, preventing the rupture from extending to the surface and resulting in a continuous distribution of seismically induced deformation. Furthermore, the significant limitation of northward displacement signifies the barrier effect of the Alashan rigid block, leading to a high strain concentration at the junction of Lajishan and Jishishan. The entire process illustrates a dynamic response chain of “rigid barrier–differential shear–rotational extrusion–fault adjustment”. This dynamic mechanism not only elucidates the rupture characteristics of the Jishishan earthquake but also reveals the unique expansion mode of the northeastern margin of the Qinghai‒Tibet Plateau through local rotation under multiple boundary constraints. This provides a crucial reference for studying tectonic evolution and strong earthquake risk at the front edge of the Himalayan collision zone.

## Discussion and conclusions

In this study, the coseismic deformation of the 2023 Jishishan earthquake (Gansu Province) was retrieved using interferometric synthetic aperture radar (InSAR) technology. Combined with source inversion and regional tectonic analysis, the key findings are as follows:

1. The coseismic deformation of the earthquake was primarily concentrated in the area bounded by the northern and southern margin faults of Lajishan, with an affected range of approximately 15 km × 25 km. The vertical displacement was significant, and both the ascending and the descending track deformation fields exhibited good spatial continuity, indicating that the fault rupture did not reach the surface.
2. Constrained by InSAR deformation data, the source inversion results confirmed that this earthquake was a typical shallow-source thrust event. Fault rupture was concentrated at subsurface depths of 0–15 km (optimally fitted source depth of 11.4 km), with a main rupture zone length of approximately 15.6 km. The fault has a strike of 303.5°, a dip angle of 49.6°, and a maximum slip of 0.70 m.
3. This earthquake represents a seismogenic structural response to regional stress adjustments during the northeastward expansion of the Qinghai‒Tibet Plateau. The main seismogenic fault is located in the southern segment of the northern margin fault of Lajishan, with thrust-dominated movement accompanied by a minor dextral compressional component. Its geological significance lies in revealing the deformation adjustment mechanism in the structural intersection area of Lajishan, the Yellow River, and Jishishan, providing a key basis for the assessment of the regional seismogenic environment.

The Okada model adopted in the core of the inversion defaults to the assumption of a homogeneous medium. Although this limitation is indirectly compensated for by the 3D coseismic deformation field forward model, the model does not directly incorporate 3D velocity structure data, making it difficult to completely eliminate the impact of medium heterogeneity on the inversion results. In the future, we intend to further improve the scientific understanding of the seismogenic mechanism and risk assessment of strong earthquakes on the northeastern margin of the Qinghai‒Tibet Plateau by integrating multisource observation data, incorporating a 3D medium heterogeneity model, and expanding the long-term deformation analysis.

## Data Availability

The Interferometric Synthetic Aperture Radar (InSAR) data used in this article were collected by the Sentinel-1 satellites operated by the European Space Agency (ESA).These data are freely available at the Sentinel Data Hub (https://dataspace.copernicus.eu/) and can also be accessed from the Alaska Satellite Facility(https://search.asf.alaska.edu/) ;The corresponding orbit product is available at the Alaska Satellite Facility (https://s1qc.asf.alaska.edu/aux_poeorb/);The initial source parameters for earthquake deformation modeling are taken from the source mechanism released by the Global Centroid Moment Tensor Project (https://www.globalcmt.org/CMTsearch.html).
